# Use of Electronic Nicotine Delivery Systems or Cigarette Smoking After US Food and Drug Administration–Prioritized Enforcement Against Fruit-Flavored Cartridges

**DOI:** 10.1001/jamanetworkopen.2023.21109

**Published:** 2023-06-30

**Authors:** Karin A. Kasza, Cheryl Rivard, Young Sik Seo, Jessica L. Reid, Shannon Gravely, Geoffrey T. Fong, David Hammond, Andrew Hyland

**Affiliations:** 1 Department of Health Behavior, Roswell Park Comprehensive Cancer Center, Buffalo, New York; 2 School of Public Health Sciences, University of Waterloo, Waterloo, Ontario, Canada; 3 Department of Psychology, University of Waterloo, Waterloo, Ontario, Canada; 4 Ontario Institute for Cancer Research, Toronto, Ontario, Canada

## Abstract

**Importance:**

The Center for Tobacco Products (CTP) of the US Food and Drug Administration prioritized its enforcement efforts against non–tobacco-, non–menthol- (fruit-) flavored cartridge electronic nicotine delivery systems (ENDS) in February 2020.

**Objective:**

To evaluate adults’ use of ENDS and cigarette smoking following the CTP’s prioritized enforcement efforts against fruit-flavored cartridge ENDS.

**Design, Setting, and Participants:**

In this population-based, nationally representative US cohort study, data were collected from the Population Assessment of Tobacco and Health Study from December 2018 to November 2019 (hereafter referred to as 2019) and/or from September 2020 to December 2020 (Adult Telephone Survey, hereafter referred to as 2020). Adults (aged ≥21 years) who used ENDS in the past 30 days and smoked cigarettes in the past 30 days or quit smoking cigarettes in the past year (n = 3173) were evaluated. Data were analyzed from January 1, 2022, to May 2, 2023.

**Exposure:**

ENDS flavor-device combinations used.

**Main Outcomes and Measures:**

Outcome measures were cross-sectional prevalence of ENDS flavor-device combinations used in 2019 (n = 2654) and 2020 (n = 519) and longitudinal transitions in cigarette smoking (cessation [no smoking in the past 30 days in 2020 among those who smoked in 2019; n = 876] and relapse [smoking in the past 30 days in 2020 among those who recently quit in 2019; n = 137]) as a function of ENDS flavor-device combination used in 2019.

**Results:**

The sample in 2019 included 2654 individuals (55% male [95% CI, 53%-58%]). Among those who used ENDS and smoked cigarettes, fruit-flavored cartridge ENDS use decreased from 13.9% (95% CI, 12.1%-15.9%) in 2019 to 7.9% (95% CI, 5.1%-12.1%) in 2020 (*P* = .01), whereas fruit-flavored disposable ENDS use increased from 4.0% (95% CI, 3.1%-5.1%) in 2019 to 14.5% (95% CI, 11.6%-18.0%) in 2020 (*P* < .001). Patterns were similar among those who recently quit smoking. Neither cigarette cessation nor relapse rates differed between those who used ENDS that were vs were not prioritized for enforcement efforts (cessation: 23.4% [95% CI, 18.1%-29.7%] vs 26.4% [95% CI, 22.4%-30.8%]; adjusted odds ratio, 1.12; 95% CI, 0.57-2.21; relapse: 32.7% [95% CI, 17.1%-53.4%] vs 29.8% [95% CI, 20.3%-41.3%]; adjusted odds ratio, 0.96; 95% CI, 0.24-3.84).

**Conclusions and Relevance:**

In this nationally representative US cohort study of adults who smoked cigarettes and used ENDS, fruit-flavored cartridge ENDS use was nearly halved between 2019 and 2020. Cigarette cessation and relapse rates did not differ between those who used ENDS targeted by CTP and those who used other ENDS.

## Key Points

**Question:**

Did adults’ use of electronic nicotine delivery systems (ENDS) and cigarette smoking behaviors change after the Center for Tobacco Products (CTP) of the US Food and Drug Administration prioritized enforcement efforts against fruit-flavored cartridge ENDS in February 2020?

**Findings:**

This nationally representative US cohort study found a significant decrease in fruit-flavored cartridge ENDS use among adults in 2020; no differences were found in cigarette cessation or relapse rates between those who used ENDS that were vs were not targeted by the CTP.

**Meaning:**

Findings provide no indication that the CTP’s 2020 targeted ENDS enforcement priority was associated with reduced smoking cessation or increased smoking relapse among adults who used ENDS.

## Teaser

This cohort study evaluates the use of electronic nicotine delivery systems (ENDS) and cigarette smoking after prioritized enforcement efforts against the use of fruit-flavored nicotine cartridges.

## Introduction

The Center for Tobacco Products (CTP) of the US Food and Drug Administration (FDA) published *Enforcement Priorities for Electronic Nicotine Delivery Systems (ENDS) and Other Deemed Products on the Market Without Premarket Authorization*^1^ in January 2020. In this report, the FDA prioritized its enforcement efforts against “any flavored, cartridge-based ENDS product (other than a tobacco- or menthol-flavored ENDS product),” which came into effect in February 2020. These priorities were part of a suite of actions intended to reduce ENDS use among young people, which had been rapidly increasing.^2,3,4,5,6^ In particular, JUUL, a discreet, pod-style ENDS device that uses replaceable cartridges containing a nicotine salt–based formulation, quickly increased in popularity and became the leading ENDS brand on the US market by 2017.^7^ JUUL pods were initially available in fruity and sweet flavors, such as fruit medley, mango, vanilla, and crème brulée, although in November 2019, JUUL removed all flavors other than menthol and tobacco flavors from the US market.^8^

Limiting the availability of fruit- or sweet-flavored ENDS may reduce the appeal of ENDS among youth; however, it may also reduce ENDS appeal among adults because most adults who use ENDS also use non–tobacco-flavored ENDS.^9^ A key concern with ENDS flavor and device restrictions is whether adults who smoke cigarettes will be hindered in quitting smoking and whether adults who have quit cigarette smoking and use ENDS may relapse to smoking if their preferred ENDS products are no longer available.^10,11,12,13^ However, empirical data on real-world behavior change to support or refute these concerns are limited. A cross-sectional online survey conducted in the US in 2021 (1 year after CTP’s ENDS enforcement priorities became effective) evaluated ENDS flavor use among those who used ENDS in a recent cigarette quit attempt and found no difference in self-report of quitting smoking because of experiencing ENDS flavor restrictions, no difference in quitting because of preferring tobacco or menthol flavor vs another flavor, and no difference in switching ENDS flavors because of experiencing flavor restrictions.^14^

To date, only 1 nationally representative, longitudinal study has evaluated use of ENDS flavors and device types among adults who used ENDS (at least weekly) and who currently or formerly smoked cigarettes before and after the effective date of the CTP’s enforcement priorities; Gravely et al^15^ analyzed data from US adults (aged ≥18 years) who participated in the web-based International Tobacco Control (ITC) Four Country Smoking and Vaping Surveys conducted from February to July 2018 and from February to June 2020, which was during the first few months after the effective date of CTP’s enforcement priorities. The 2020 survey also coincided with the onset of the COVID-19 pandemic in the US, which caused large-scale shutdowns across the nation.^16^ Gravely et al^15^ found that more than half of adults who smoked cigarettes and vaped non–tobacco-, non–menthol- (hereafter referred to as fruit-) flavored cartridge ENDS products in 2018 switched to vaping an ENDS flavor-device combination in 2020 that was not prioritized for CTP’s enforcement efforts. In contrast, among adults who smoked cigarettes and vaped an ENDS flavor-device combination other than fruit-flavored cartridge ENDS in 2018, only 6% switched to vaping fruit-flavored cartridge ENDS in 2020. These results are consistent with the hypothesis that CTP’s enforcement priorities may have impacted the vaping behaviors of adults who smoke cigarettes, although the ITC analyses were underpowered to assess potential effects on smoking behaviors, and follow-up data collection was limited to the first 5 months of the enforcement priority implementation in 2020. To address current gaps in the literature, we evaluated adults’ use of ENDS and cigarette smoking after the CTP’s prioritized enforcement efforts against fruit-flavored cartridge ENDS.

## Methods

### Participants

In this cohort study, we analyzed data from adults 21 years or older who participated in the nationally representative, longitudinal Population Assessment of Tobacco and Health (PATH) Study from December 2018 to November 2019 (wave 5, hereafter referred to as 2019) and/or from September 2020 to December 2020 (Adult Telephone Survey, hereafter referred to as 2020). The 2019 data were collected using in-person, audio, computer-assisted self-interviews. Because of the COVID-19 pandemic, the 2020 data were collected by telephone only.^17^ The PATH Study was conducted by Westat and approved by the Westat Institutional Review Board; the study reported here was approved by the Roswell Park Institutional Review Board. All adults provided written informed consent. This study follows the Strengthening the Reporting of Observational Studies in Epidemiology (STROBE) reporting guideline for cohort studies.

The response rates were 88% in 2019 and 56% in 2020, and Nonresponse Bias Analysis Reports have been published for each wave of the PATH Study.^17^ Compared with earlier waves, there were differences in the 2020 response rates by socioeconomic status, race and ethnicity, and tobacco use status, similar to other government-sponsored studies conducted during COVID. Importantly, the weighting adjustments were found to essentially eliminate this underrepresentation or overrepresentation, leaving little remaining potential for nonresponse bias in the 2020 estimates.^17^ Further details on the PATH Study design and methods^18,19,20^ and demographic and tobacco use distributions^21^ are published elsewhere. Details on interviewing procedures, questionnaires, sampling, weighting, response rates, and accessing the data are available from the National Addiction & HIV Data Archive Program.^17^

Our analytic sample was composed of adults who used ENDS and currently or formerly smoked cigarettes in 2019 (n = 2654) or in 2020 (n = 519). Longitudinal analyses were among 1013 adults who used ENDS and currently or formerly smoked cigarettes in 2019 and who participated in 2020. Among those who used ENDS and who currently or recently smoked cigarettes, we evaluated differences in the prevalence of ENDS flavor-device combinations used in 2020 compared with 2019. Among those who used ENDS and smoked cigarettes in the past 30 days in 2019, we evaluated smoking cessation in 2020 as a function of ENDS flavor-device combinations used in 2019, and among those who used ENDS and recently smoked cigarettes in 2019, we evaluated smoking relapse as a function of ENDS flavor-device combinations used in 2019. Estimates were weighted to adjust for the PATH Study’s complex study design characteristics (eg, oversampling) and attrition so that they represent the resident population of the US in 2019 and 2020 who were in the civilian, noninstitutionalized population in 2016 and 2017, as described in detail elsewhere.^17^

### Measures

#### ENDS Flavor and Device Type Used (Assessed in 2019 and 2020)

At each interview, respondents were asked whether they used ENDS (ie, e-cigarettes, e-cigars, e-pipes, and e-hookahs) in the past 30 days, and those who had were asked whether they currently use ENDS every day, some days, or not at all. ENDS users were asked, “What flavor is [your regular brand/the brand you last used]? Choose all that apply.” Response options were tobacco-flavored; menthol or mint; clove or spice; fruit; chocolate; an alcoholic drink (such as wine, cognac, margarita, or other cocktails); a nonalcoholic drink (such as coffee, soda, energy drinks, or other beverages); candy, desserts, or other sweets; and some other flavor. We categorized ENDS flavor into the following 4 mutually exclusive categories: (1) only tobacco flavor, (2) only menthol or mint flavor, (3) only nontobacco, nonmenthol, and nonmint flavor(s) (ie, any flavor except for tobacco or menthol or mint), referred to throughout as fruit; and (4) multiple flavors (ie, any combination of tobacco, menthol or mint, and/or fruit flavors).

Those who used ENDS were also asked what kind of ENDS product they use most often (a disposable device, a device that uses replaceable prefilled cartridges, a device with a tank that you refill with liquids, a mod system, or something else). We categorized ENDS device types into the following 3 mutually exclusive categories: (1) disposable product (ie, not rechargeable), (2) cartridge product (ie, rechargeable and uses cartridges), and (3) tank or mod system (ie, rechargeable and does not use cartridges); 1% of those who used ENDS were excluded from analysis because of having an ENDS device type that could not be categorized.

We created a 12-level ENDS flavor-device type combination variable as follows: (1) tobacco flavor, disposable; (2) menthol or mint flavor, disposable; (3) fruit flavor, disposable; (4) multiple flavors, disposable; (5) tobacco flavor, cartridge; (6) menthol or mint flavor, cartridge; (7) fruit flavor, cartridge; (8) multiple flavors, cartridge; (9) tobacco flavor, tank or mod; (10) menthol or mint flavor, tank or mod; (11) fruit flavor, tank or mod; and (12) multiple flavors, tank or mod. We also combined these categories into a dichotomous variable to indicate whether the combination was prioritized for CTP enforcement efforts (ie, fruit-flavored cartridge vs all other combinations).

#### Cigarette Smoking Status (Assessed in 2019 and 2020)

At each interview, respondents were asked if they smoked cigarettes in the past 12 months and if they smoked cigarettes in the past 30 days. Cigarette smoking status was defined as current smoking if a respondent smoked cigarettes in the past 30 days. Cigarette smoking status was defined as former cigarette smoking if a respondent smoked cigarettes in the past 12 months but did not smoke cigarettes in the past 30 days.

#### Cigarette Cessation or Cigarette Relapse (Assessed in 2020)

Cigarette cessation was defined as no cigarette smoking in the past 30 days in 2020 among those who smoked cigarettes in the past 30 days when interviewed in 2019 (ie, past 30-day cigarette smoking in 2019 to no past 30-day cigarette smoking in 2020). Cigarette relapse was defined as cigarette smoking in the past 30 days in 2020 among those who did not smoke cigarettes in the past 30 days but did smoke cigarettes in the past 12 months when interviewed in 2019 (ie, no past 30-day cigarette smoking in 2019 [although smoked within past year in 2019] to past 30-day smoking in 2020).

#### Other Measures Evaluated as Covariates

Respondents also reported their age, biological sex (male or female), race (White or another race [ie, Black/African American, American Indian or Alaska Native, Asian Indian, Chinese, Filipino, Japanese, Korean, Vietnamese, Other Asian, Native Hawaiian, Guamanian or Chamorro, Samoan, or other Pacific Islander]), ethnicity (Hispanic or not Hispanic), income (<$10 000, $10 000-$24 999, $25 000-$49 999, $50 000-$99 999, or ≥$100 000), educational level (no college, some college or associate’s degree, or bachelor’s degree or higher), frequency of cigarette smoking (daily or nondaily), cigarettes smoked per month, menthol vs nonmenthol cigarette smoking, and frequency of ENDS use (daily or nondaily). We included race and ethnicity as covariates in this study because they are known to be correlated with tobacco use. Racial and ethnic groups were combined to protect participant confidentiality.

### Statistical Analysis

#### Cross-Sectional Comparisons

We evaluated the cross-sectional prevalence of ENDS flavor-device combination used in 2019 and 2020 among adults who used ENDS in the past 30 days, stratified by cigarette smoking status (current vs former). We used χ^2^ tests to determine whether the prevalence of each ENDS flavor-device combination differed between 2019 and 2020, overall and stratified by cigarette smoking status. An α = .05 was considered statistically significant. The 2019 estimates were weighted using the 2019 single-wave weights for the 2016-2017 cohort, and the 2020 estimates were weighted using the 2020 all-waves weights for the 2016-2017 cohort. We estimated variances using the balanced repeated replication method^22^ with Fay adjustment set to 0.3 to increase estimate stability.^23^ Data were analyzed from January 1, 2022, to May 2, 2023.

#### Longitudinal Transitions

We evaluated within-person transitions in cigarette smoking between 2019 and 2020 among adults who used ENDS in the past 30 days in 2019 as a function of whether the ENDS flavor-device combination used in 2019 was prioritized for CTP’s enforcement efforts in 2020 (ie, fruit-flavored cartridge ENDS vs all other ENDS flavor-device categories). These analyses were conducted separately among those who smoked cigarettes in 2019 (to evaluate cigarette cessation in 2020) and among those who formerly smoked cigarettes in 2019 (to evaluate cigarette relapse in 2020). Separate logistic regression analyses evaluating cigarette cessation and cigarette relapse were conducted unadjusted and adjusted for covariates (all assessed in 2019), with α < .05 considered statistically significant. All longitudinal transition analyses were weighted using the 2020 all-waves weights for the 2016-2017 cohort. Variances were estimated using the balanced repeated replication method^22^ with Fay adjustment set to 0.3 to increase estimate stability.^23^

## Results

The population in 2019 included 2654 individuals (55% male [95% CI, 53%-58%] and 45% female [95% CI, 42%-47%]; 78% White [95% CI, 76%-80%] and 22% other race [95% CI, 20%-24%]; and 14% Hispanic [95% CI, 12%-16%] and 86% not Hispanic [95% CI, 84%-88%]). Of the 2019 population, 53% were aged 21 to 34 years (95% CI, 51%-56%), 35% were aged 35 to 54 years (95% CI, 33%-37%), and 12% were 55 years or older (95% CI, 10%-13%). Of this population, 65% had a household income less than $50 000 (95% CI, 62%-67%), 46% had no college education (95% CI, 44%-49%), 39% had some college or associate’s degree (95% CI, 37%-41%), and 15% had a bachelor’s degree or higher (95% CI, 13%-17%).

### Prevalence of ENDS Flavor-Device Combinations Used in 2019 and 2020

**Figure 1.**
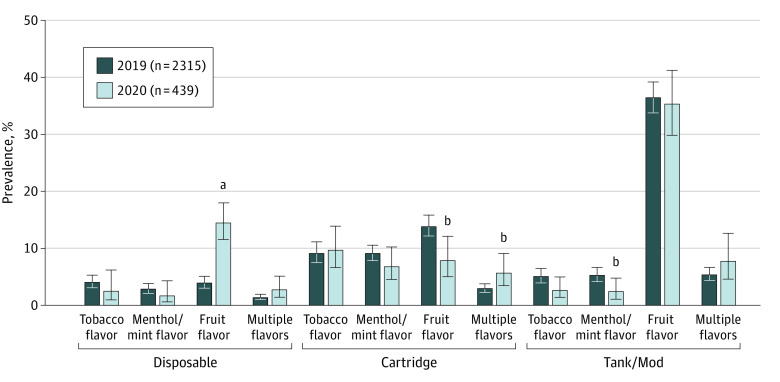
Electronic Nicotine Delivery Systems (ENDS) Flavor-Device Combinations Used Among US Adults Who Smoked Cigarettes and Used ENDS, 2019 and 2020 Error bars indicate 95% CIs. ^a^*P* < .001 for 2020 estimate compared with 2019 estimate. ^b^*P* < .05 for 2020 estimate compared with 2019 estimate.

Figure 1 shows the distribution of ENDS flavor-device combinations used among those who used ENDS and who smoked cigarettes. The fraction of this group who used fruit-flavored cartridge ENDS significantly decreased from 13.9% in 2019 (95% CI, 12.1%-15.9%) to 7.9% in 2020 (95% CI, 5.1%-12.1%; *P* = .01), as did the fraction that used menthol- or mint-flavored tank or mod ENDS (from 5.3% [95% CI, 4.2%-6.7%] to 2.5% [95% CI, 1.2%-4.9%], *P* = .03), whereas the fraction who used fruit-flavored disposable ENDS significantly increased from 4.0% (95% CI, 3.1%-5.1%) to 14.5% (95% CI, 11.6%-18.0%, *P* < .001), as did the fraction who used multiple-flavored cartridge ENDS (from 3.0% [95% CI, 2.3%-3.8%] to 5.7% [95% CI, 3.5%-9.1%], *P* = .02).

**Figure 2.**
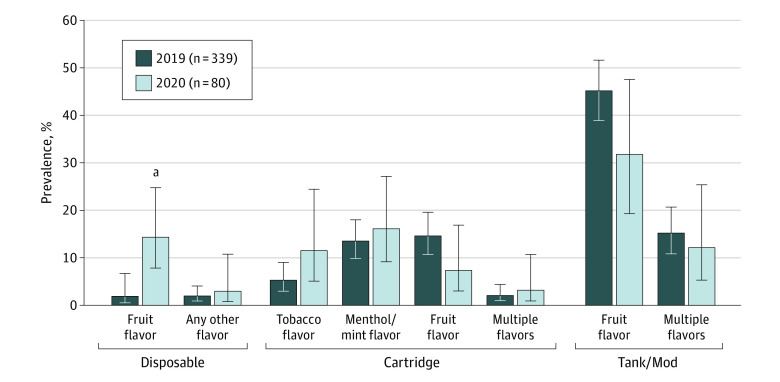
Electronic Nicotine Delivery Systems (ENDS) Flavor-Device Combinations Used Among US Adults Who Recently Quit Smoking Cigarettes and Used ENDS, 2019 and 2020 Some categories were combined because of small sample sizes. Error bars indicate 95% CIs. ^a^*P* < .001 for 2020 estimate compared with 2019 estimate.

Figure 2 shows the distribution of ENDS flavor-device combinations used among those who used ENDS and who recently quit smoking cigarettes. The proportion of this group using fruit-flavored cartridge ENDS was 14.7% in 2019 (95% CI, 10.7%-19.7%) and 7.4% in 2020 (95% CI, 3.0%-16.9%, *P* = .11), whereas the fraction who used fruit-flavored disposable ENDS significantly increased from 1.9% (95% CI, 0.5%-6.7%) to 14.4% (95% CI, 7.9%-24.9%; *P* < .001).

### Within-Person Transitions in Cigarette Smoking as a Function of ENDS Flavor-Device Combinations Used in 2019

Among those who used ENDS and who smoked cigarettes in 2019 (n = 876), 25.3% quit cigarette smoking in 2020 (95% CI, 21.9%-29.0%) (Table). Cigarette cessation rates were similar between those who used fruit-flavored cartridge ENDS (23.4% quit smoking; 95% CI, 18.1%-29.7%) and those who used any other flavor-device combination ENDS in 2019 (26.4% quit smoking; 95% CI, 22.4%-30.8%; adjusted odds ratio, 1.12; 95% CI, 0.57-2.21) (Table).

**Table.** Longitudinal Transitions in Cigarette Smoking as a Function of ENDS Flavor-Device Categories Used Among Adults Who Used ENDS in 2019^a^

ENDS flavor-device combination use in 2019	No./total No. (%) [95% CI]	AOR (95% CI)
**Cigarette smoking cessation in 2020 (past 30-d cigarette smoking in 2019 to no past 30-d cigarette smoking in 2020)** ^b^		
Overall	226/876 (25.3) [21.9-29.0]	NA
Fruit-flavored cartridge	71/313 (23.4) [18.1-29.7]	1.00 [Reference]
All other combinations	155/563 (26.4) [22.4-30.8]	1.12 (0.57-2.21)
**Cigarette smoking relapse in 2020 (no past 30-d cigarette smoking in 2019 [although smoked within past year in 2019] to past 30-d smoking in 2020)** ^c^		
Overall	39/137 (30.5) [22.6-39.7]	NA
Fruit-flavored cartridge	11/36 (32.7) [17.1-53.4]^d^	1.00 [Reference]
All other combinations	28/101 (29.8) [20.3-41.3]	0.96 (0.24-3.84)

Abbreviations: AOR, adjusted odds ratio; ENDS, electronic nicotine delivery systems; NA, not applicable.

^a^ Numbers are unweighted numbers of observations. Percentages, 95% CIs, and AORs are weighted.

^b^ Logistic regression analysis adjusted for age, sex, race, ethnicity, income, educational level, frequency of cigarette smoking, log-transformed cigarettes smoked per month, cigarette flavor, and frequency of e-cigarette use.

^c^ Logistic regression analysis adjusted for age, sex, race, ethnicity, income, educational level, and frequency of e-cigarette use.

^d^ Estimate should be interpreted with caution because it has a relative SE greater than 30 or a denominator less than 50.

Among those who used ENDS and who recently quit smoking cigarettes in 2019 (n = 137), 30.5% returned to smoking cigarettes in 2020 (95% CI, 22.6%-39.7%) (Table). Cigarette relapse rates were statistically indistinguishable between those who used fruit-flavored cartridge ENDS in 2019 (32.7% relapsed to smoking; 95% CI, 17.1%-53.4%) and those who used any other flavor-device combination ENDS in 2019 (29.8% relapsed to smoking; 95% CI, 20.3%-41.3%; adjusted odds ratio, 0.96; 95% CI, 0.24-3.84) (Table).

## Discussion

Concerns have been raised that CTP’s prioritized enforcement efforts against fruit-flavored cartridge ENDS, intended to reduce ENDS use in youth, may negatively impact adults who use ENDS and smoke cigarettes or who recently quit smoking cigarettes.^10,11,12,13^ Our nationally representative findings show a significant decrease in the prevalence of use of fruit-flavored cartridge ENDS among adults who used ENDS in 2020 compared with 2019, alongside a significant increase in the prevalence of use of fruit-flavored disposable ENDS, with the overall use of fruit-flavored ENDS being similar in each year. Our analysis yielded no evidence of reduced cigarette cessation rates or increased cigarette relapse rates among those whose ENDS products were targeted by CTP’s enforcement priorities compared with those who used ENDS products that were excluded from CTP’s priorities.

This study is the first, to our knowledge, to show an increase in the use of flavored disposable ENDS among adults in the US who currently or recently smoked cigarettes, consistent with the hypothesis that these ENDS users may have switched their device type to maintain their flavor use, which has thus far only been shown among youth in the US.^24^ It is therefore possible that shifts in adults’ use of ENDS may have contributed to the market share increase in disposable ENDS brands in 2020, such as Puff Bar, which received warning letters from the CTP in July 2020.^25^

At the same time, our findings show that the potential reach among adults of CTP’s February 2020 enforcement priorities was low. That is, in 2019, only 14% of adults who used ENDS (and who currently or formerly smoked cigarettes) were using fruit-flavored cartridge ENDS, meaning that only approximately 1 in 7 adults were using the specific flavor-device combination that was targeted by the CTP. The low reach and low potential for impact on adults aligns with the intended goal of CTP’s actions to impact youth while considering the whole population when making tobacco regulatory decisions. Although our findings show some ENDS flavor-device product switching among adults and some changes in the national prevalence of ENDS flavor-device combinations used among adults before and after CTP’s enforcement priorities, use of fruit-flavored tank or mod devices remain by far the most common combination of ENDS flavor and device used by adults who smoke cigarettes in the US and the most common combination used by adults who recently quit smoking cigarettes in the US.

### Limitations

Although we found no differences in cigarette cessation rates or relapse rates between those who used ENDS that were prioritized for enforcement efforts and those who used other ENDS, we note that sample sizes were small for evaluating transitions in cigarette smoking owing in part to the small segment of adults in the US who were using the targeted ENDS products. Another limitation of this study was including a range of ENDS use and smoking frequencies, and results may differ for those who used ENDS more frequently and/or who may be more dependent on nicotine (sample sizes precluded us from further stratifying analyses). We were also unable to distinguish between menthol-flavored ENDS and mint-flavored ENDS because these flavors were combined when participants were queried in 2019. Additionally, 2020 follow-up data were collected within the year after CTP’s enforcement priorities, which was also during the COVID-19 pandemic. Thus, we cannot necessarily attribute observed changes in behavior to the enforcement priorities per se, and analysis of subsequent waves of PATH Study data is needed to evaluate behavior changes in the longer term. Lastly, we evaluated adults 21 years and older, and findings may not be generalizable to those younger than 21 years.

## Conclusions

The CTP’s February 2020 ENDS enforcement priority to target its enforcement efforts against fruit-flavored cartridge ENDS had low reach among adults who used ENDS and currently or recently smoked cigarettes, consistent with the goal of prioritizing the reduction of youth ENDS use while limiting potential negative effects on other segments of the population. Our findings reveal national-level changes in the ENDS flavor-device combinations used among adults who smoke in the US before and after CTP’s ENDS enforcement priorities, with adults shifting from use of flavored cartridge products to use of flavored disposable products. We found no evidence to support concerns that adults’ cigarette smoking behaviors will be negatively impacted by this effort to protect youth.

## Notes

Supplement.
Data Sharing Statement
