# Supplementary material for: Use of Electronic Nicotine Delivery Systems or Cigarette Smoking After US Food and Drug Administration–Prioritized Enforcement Against Fruit-Flavored Cartridges
Source: JAMA Netw Open. 2023 Jun 30;6(6):e2321109. doi: 10.1001/jamanetworkopen.2023.21109 (PMC10314301; doi:10.1001/jamanetworkopen.2023.21109)
Supplement: Supplement. — Data Sharing Statement [file jamanetwopen-e2321109-s001.pdf]

Data Sharing Statement

Kasza. Use of Electronic Nicotine Delivery Systems or Cigarette Smoking After US Food and Drug Administration–Prioritized Enforcement Against Fruit-Flavored Cartridges. *JAMA Netw Open*. Published June 30, 2023. doi:10.1001/jamanetworkopen.2023.21109

Data
Data available: Yes
Data types: Other (please specify)
Additional Information: Data from the PATH Study may be obtained from a third party and are not publicly available (https://www.icpsr.umich.edu/icpsrweb/NAHDAP/studies/36231). Application instructions and conditions of use are available at the website previously mentioned.
How to access data: Data from the PATH Study may be obtained from a third party and are not publicly available (https://www.icpsr.umich.edu/icpsrweb/NAHDAP/studies/36231). Application instructions and conditions of use are available at the website previously mentioned.
When available: With publication

Supporting Documents
Document types: Other (please specify)
Additional Information: Data from the PATH Study may be obtained from a third party and are not publicly available (https://www.icpsr.umich.edu/icpsrweb/NAHDAP/studies/36231). Application instructions and conditions of use are available at the website previously mentioned.
How to access documents: Data from the PATH Study may be obtained from a third party and are not publicly available (https://www.icpsr.umich.edu/icpsrweb/NAHDAP/studies/36231). Application instructions and conditions of use are available at the website previously mentioned.
When available: With publication

Additional Information
Who can access the data: Data from the PATH Study may be obtained from a third party and are not publicly available (https://www.icpsr.umich.edu/icpsrweb/NAHDAP/studies/36231). Application instructions and conditions of use are available at the website previously mentioned.
Types of analyses: Data from the PATH Study may be obtained from a third party and are not publicly available (https://www.icpsr.umich.edu/icpsrweb/NAHDAP/studies/36231). Application instructions and conditions of use are available at the website previously mentioned.
Mechanisms of data availability: Data from the PATH Study may be obtained from a third party and are not publicly available (https://www.icpsr.umich.edu/icpsrweb/NAHDAP/studies/36231). Application instructions and conditions of use are available at the website previously mentioned.
Any additional restrictions: Data from the PATH Study may be obtained from a third party and are not publicly available (https://www.icpsr.umich.edu/icpsrweb/NAHDAP/studies/36231). Application instructions and conditions of use are available at the website previously mentioned.
